# A Rare Case of Stevens-Johnson Syndrome Secondary to Levofloxacin Administration: Presentation and Management

**DOI:** 10.7759/cureus.106479

**Published:** 2026-04-05

**Authors:** Marios Chrysostomou, Nisarg Shah, Aparna Ravikumar, Fateh Khan

**Affiliations:** 1 Internal Medicine, York and Scarborough Teaching Hospitals NHS Foundation Trust, Scarborough, GBR; 2 Respiratory Medicine, York and Scarborough Teaching Hospitals NHS Foundation Trust, Scarborough, GBR

**Keywords:** antibiotic therapy, community-acquired pneumonia treatment, cutaneous adverse drug reaction, fluoroquinolones, ocular involvement, stevens-johnson syndrome, toxic epidermal necrolysis (ten)

## Abstract

Stevens-Johnson syndrome (SJS) is a rare but life-threatening mucocutaneous reaction, most commonly triggered by medications and characterised by rapid progression, extensive mucosal involvement, and risk of long-term sequelae. We describe the case of a 74-year-old woman who developed severe SJS shortly after receiving levofloxacin for community-acquired pneumonia. Within days of antibiotic exposure, she experienced extensive mucocutaneous ulceration and profound ocular involvement, necessitating intensive multidisciplinary care.

This case highlights the unpredictable nature of drug-associated SJS, the challenges of managing extensive ocular disease, and the importance of vigilance when prescribing widely used antibiotics. By underscoring the value of early identification and coordinated multidisciplinary care, it serves as a reminder that even familiar medications can provoke uncommon but severe reactions with the potential to cause life-changing consequences.

## Introduction

Stevens-Johnson syndrome (SJS) and toxic epidermal necrolysis (TEN) are rare but potentially fatal type IVc hypersensitivity reactions characterised by cytotoxic T-lymphocyte-mediated epithelial injury [1]. Clinically, these conditions present with extensive erythema, erosions, and blistering involving the skin and mucous membranes, including the lips, oral cavity, eyes, and genitalia, and are often accompanied by high fever and systemic malaise [2]. SJS is defined by epidermal detachment affecting less than 10% of the total body surface area (BSA). Involvement of 10-30% BSA is classified as SJS/TEN overlap, while TEN is diagnosed when more than 30% of the BSA is affected [1].

The pathogenesis of SJS is believed to involve a complex interplay between genetic susceptibility and external triggers, most commonly medications or infections, leading to widespread keratinocyte apoptosis and epidermal necrosis. Drug exposure remains the most frequent precipitating factor for SJS [3]. A recent systematic review and meta-analysis of international databases reported that antibiotics were implicated in approximately 28% of SJS/TEN cases worldwide. Among these, sulphonamides accounted for 32%, penicillins 22%, cephalosporins 11%, fluoroquinolones 4%, and macrolides 2% [3].

The estimated global incidence of SJS/TEN is 5.76 cases per million population per year, with reported mortality rates of 4.8-9% for SJS, 19.4-29% for SJS/TEN overlap, and 14.8-48% for TEN [4,5]. Among fluoroquinolone-associated cases of SJS, those attributed specifically to levofloxacin are exceptionally rare, with only a limited number of isolated cases described in the literature [6-9].

This case report describes a rare presentation of SJS likely secondary to levofloxacin, a commonly prescribed fluoroquinolone antibiotic. Given that levofloxacin features prominently in local UK hospital guidelines for the management of community-acquired pneumonia [10,11], raising awareness of this uncommon yet life-threatening adverse reaction is essential. Such cases serve as important educational tools, reinforcing the need for heightened clinical vigilance, prompt recognition of iatrogenic drug reactions, and timely consideration of alternative therapies. This report also highlights the critical role of early drug withdrawal and multidisciplinary management, as delayed intervention may result in severe complications, including permanent visual impairment, multisystem involvement, and mortality.

## Case presentation

**Figure 1:**
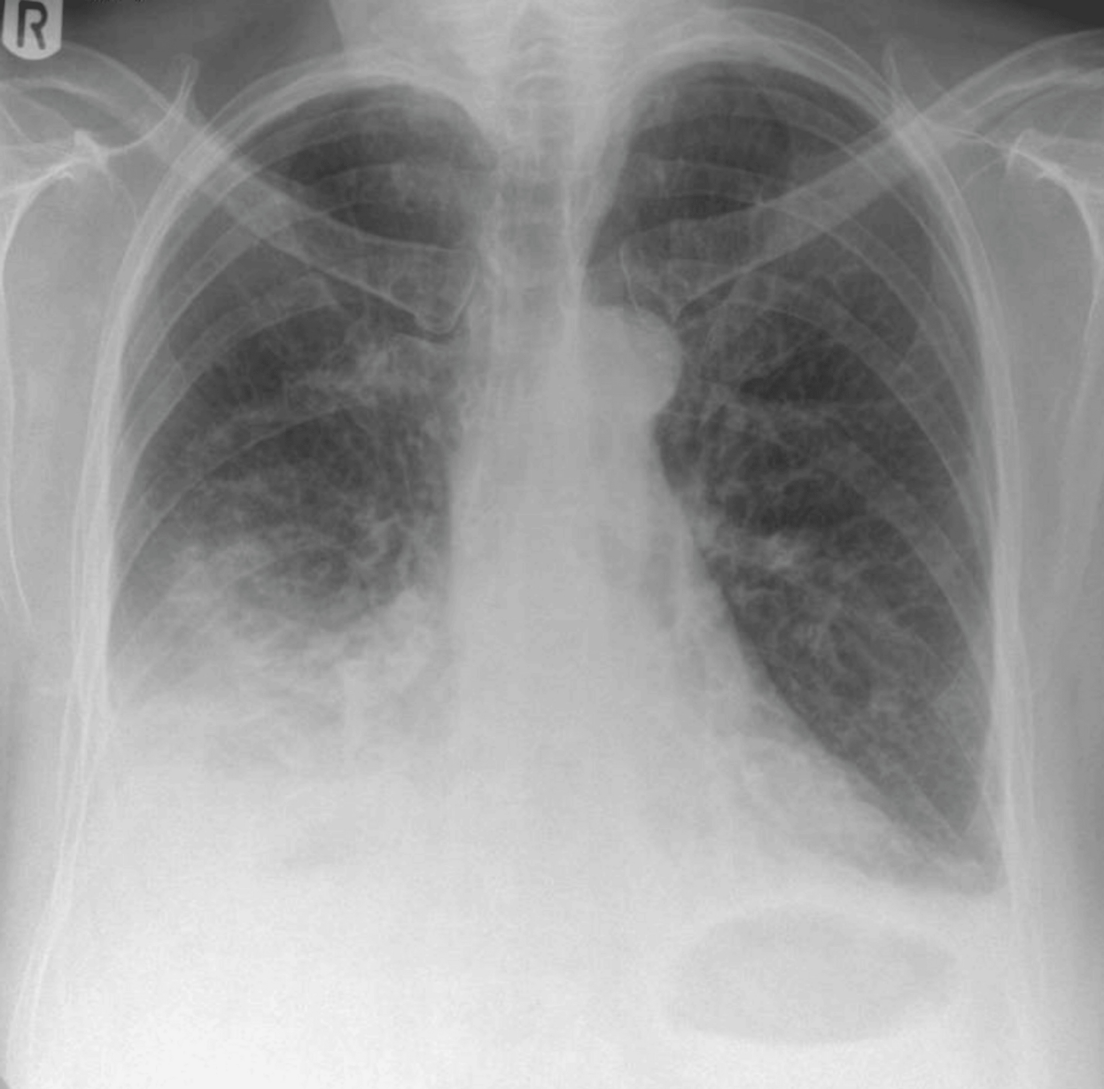
Chest radiograph demonstrating right middle and lower lobe consolidation Chest radiograph obtained at admission demonstrating dense consolidation involving the right middle and lower lobes, consistent with severe community-acquired pneumonia at initial presentation.

A 74-year-old female patient with a background of hypercholesterolaemia and penicillin allergy, not taking any regular medications prior to admission, presented with four days of worsening breathlessness and pyrexia. Admission blood tests showed elevated C-reactive protein (CRP), neutropenia, and leukopenia, consistent with severe systemic infection at presentation, as summarised in Table 1. Arterial gases confirmed type 1 respiratory failure. Chest radiography demonstrated dense consolidation in the right mid- and lower zones as shown in Figure 1, while computed tomography pulmonary angiogram (CTPA) excluded pulmonary embolism.

**Table 1:** Laboratory parameters on the day of admission

Parameter	Value	Reference range
Haemoglobin (g/L)	155	115-165
White cell count (×10⁹/L)	0.8	4.0-11.0
Neutrophils (×10⁹/L)	0.54	2.0-7.5
Lymphocytes (×10⁹/L)	0.21	1.0-4.0
Monocytes (×10⁹/L)	0.02	0.2-0.8
Eosinophils (×10⁹/L)	0.00	0.0-0.4
Basophils (×10⁹/L)	0.02	0.0-0.1
Mean corpuscular volume (fL)	93	80-100
Platelets (×10⁹/L)	316	150-400
C-reactive protein (mg/L)	459	<5
Creatinine (µmol/L)	75	45-90
Urea (mmol/L)	12.4	2.5-7.8
Estimated glomerular filtration rate (mL/min/1.73 m²)	67	>60
Sodium (mmol/L)	133	135-145
Potassium (mmol/L)	5.3	3.5-5.0
Bicarbonate (mmol/L)	17.6	22-28
Magnesium (mmol/L)	0.75	0.70-1.00
Adjusted calcium (mmol/L)	2.52	2.20-2.60
Phosphate (mmol/L)	0.86	0.80-1.50
Albumin (g/L)	33	35-50
Total protein (g/L)	76	60-80
Alanine aminotransferase (IU/L)	35	<40
Alkaline phosphatase (IU/L)	58	30-130
Bilirubin (µmol/L)	12	<21
Prothrombin time (s)	15.9	11-14
Activated partial thromboplastin time (s)	28.8	25-35
Fibrinogen (g/L)	>4.5	1.5-4.5
B-type natriuretic peptide (pg/mL)	672	<100

The patient required admission to the intensive care unit due to worsening hypoxic respiratory failure and was started on high-flow nasal oxygen with close monitoring, with the possibility of intubation if further deterioration occurred. Blood cultures were positive for *Streptococcus pneumoniae*, and intravenous levofloxacin for severe community-acquired pneumonia was commenced in accordance with local guidelines as an alternative antibiotic in patients with penicillin allergy.

Notably, neutropenia was present at admission prior to the initiation of levofloxacin. This was considered multifactorial, likely reflecting the effects of severe infection, physiological stress, and immune dysregulation. Neutrophil counts demonstrated rapid recovery within 48 hours, supporting a sepsis-related aetiology. The haematological trend throughout admission is summarised in Table 2.

**Table 2:** Haematological trend throughout the clinical course and follow-up Levofloxacin was initiated after admission when neutropenia was already present. Neutrophil counts normalised within 48 hours, consistent with transient sepsis-associated neutropenia. Linezolid was commenced subsequently, at a time when neutrophil and platelet counts had recovered. Haematological parameters were monitored throughout admission, with complete recovery observed at the three-month follow-up.

Parameter	Admission (pre-levofloxacin)	24 hours	48 hours	72 hours	96 hours (at linezolid initiation)	Discharge (day 21)	3-month follow-up	Reference range
Haemoglobin (g/L)	155	130	140	131	114	93	134	115-165
White cell count (×10⁹/L)	0.5	2.9	13.1	25.3	12.5	15.3	6.73	4.0-11.0
Neutrophils (×10⁹/L)	0.39	2.54	12.28	23.85	10.79	12.83	4.29	2.0-7.5
Platelets (×10⁹/L)	316	182	185	180	352	584	391	150-400
C-reactive protein (mg/L)	459	420	276	258	79	172	4	<5

In addition to levofloxacin, only paracetamol, salbutamol nebulisers, and prophylactic enoxaparin were administered during the early phase of hospitalisation. The patient also denied use of over-the-counter medications, herbal remedies, or recent contrast exposure.

Within 72 hours of the first levofloxacin dose, she developed abrupt lip swelling. Given the need for continued antimicrobial coverage in the setting of severe pneumococcal infection and documented penicillin allergy, antimicrobial therapy was escalated to linezolid following microbiology consultation. At the time of initiation, neutrophil counts had recovered with subsequent reactive neutrophilia, and platelet counts remained within the normal range. Haematological parameters were closely monitored thereafter, as summarised in Table 2.

**Figure 2:**
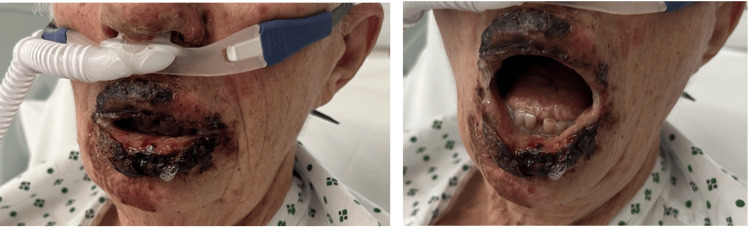
Severe mucocutaneous involvement in Stevens-Johnson syndrome Clinical photograph taken approximately one week after the initiation of levofloxacin therapy demonstrating haemorrhagic crusting, necrosis, and epidermal sloughing involving the lips and perioral region.

Over the next 48 hours, the lips and adjacent perioral skin became painful, erythematous macules evolved into flaccid bullae, and the epidermis sloughed, leaving haemorrhagic crusts extending to the nose and cheeks as demonstrated in Figure 2. Mucosal involvement of the oral cavity and both conjunctivae subsequently developed.

Dermatology confirmed a clinical diagnosis of SJS based on mucocutaneous involvement with <10% BSA detachment and multisite mucosal involvement. The clinical morphology of painful erythematous macules progressing to flaccid bullae with epidermal sloughing, together with the temporal association with recent levofloxacin exposure, was considered clinically consistent with drug-associated SJS. The severity of illness was assessed using the SCORTEN (SCORe of Toxic Epidermal Necrolysis) prognostic scoring system, with a score of 3 as summarised in Table 3, and supportive care without systemic corticosteroids was recommended.

**Table 3:** SCORTEN variables and values at presentation SCORTEN: SCORe of Toxic Epidermal Necrolysis; bpm: beats per minute; BSA: body surface area

SCORTEN variable	Patient value	Points
Age >40 years	74 years	1
Presence of malignancy	No	0
Heart rate >120 bpm	<120 bpm	0
Epidermal detachment >10% BSA	<10% BSA	0
Serum urea >10 mmol/L	12.4 mmol/L	1
Serum glucose >14 mmol/L	Within normal range	0
Serum bicarbonate <20 mmol/L	17.6 mmol/L	1
Total score		3

Given the clinical diagnosis of SJS, other potential infectious and pharmacological triggers were actively investigated and excluded. In particular, *Mycoplasma pneumoniae* infection, a recognised cause of SJS, was specifically screened for using respiratory viral polymerase chain reaction (PCR) panels, both of which returned negative. Additional investigations ruled out alternative infectious or pharmacological triggers. Stool cultures were negative for *Escherichia coli* and *Cryptosporidium*, and *Clostridioides difficile* toxin was not detected. Screening for methicillin-resistant *Staphylococcus aureus* (MRSA) was negative. Blood cultures yielded *Streptococcus pneumoniae* (serotype 23A), consistent with the diagnosis of community-acquired pneumonia. Urine cultures were sterile. A comprehensive respiratory viral panel was negative for SARS-CoV-2, influenza A/B, *Mycoplasma pneumoniae*, adenovirus, rhinovirus, enterovirus, metapneumovirus, seasonal coronaviruses (229E, NL63, OC43, HKU1), parainfluenza viruses (types 1-4), respiratory syncytial virus (A/B), and *Bordetella pertussis*. In addition, the *Legionella *urinary antigen was negative, and human immunodeficiency virus (HIV) screening (Ab/Ag) was non-reactive.

Importantly, no other concurrent pharmacological exposures were identified, supporting a likely association between levofloxacin exposure and the onset of SJS in this case.

**Figure 3:**
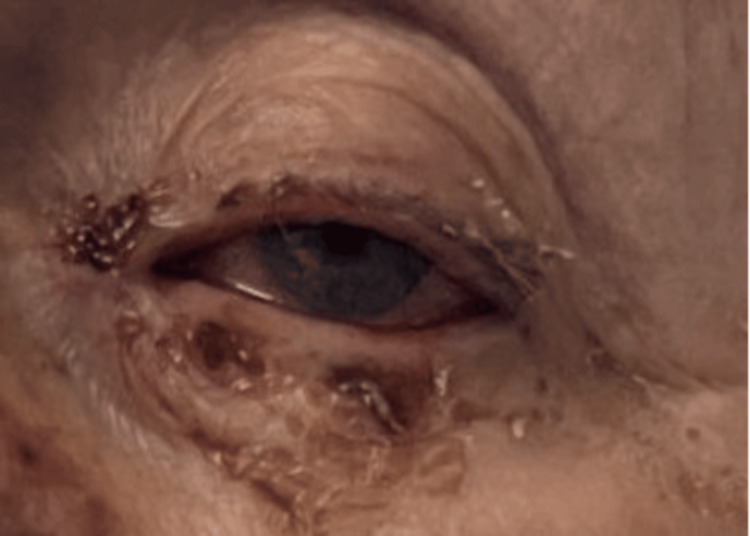
Ocular and periocular involvement in Stevens-Johnson syndrome Clinical photograph demonstrating marked eyelid erythema, periocular desquamation, conjunctival injection, and haemorrhagic crusting consistent with acute ocular surface inflammation.

On day 10, the left eye was noted to be swollen shut with purulent discharge (Figure 3), and visual acuity had declined to 6/60. Visual acuity of the right eye was 6/12. Intraocular pressures were 11.3 mmHg in the right eye (oculus dexter, OD) and 9.0 mmHg in the left eye (oculus sinister, OS). A slit-lamp examination revealed a 1.7×2 mm corneal epithelial defect in the left eye and a dendritic ulcer in the right. Conjunctival swabs confirmed herpes simplex virus (HSV)-1 positivity, and ganciclovir eye drops were added to the regimen of hourly preservative-free lubricants, topical dexamethasone 0.1%, and chloramphenicol. 

In the context of established SJS with ocular surface epithelial disruption, these findings were considered consistent with secondary HSV keratitis/reactivation.

With daily ophthalmology review, steady re-epithelialisation was observed, and by day 15 on topical treatment, both corneas had healed with improved acuity (6/12 in the right eye and 6/18 in the left eye). No scarring, fluorescein staining, or symblepharon formation was noted. As ocular healing progressed, topical steroids were gradually tapered.

Although amniotic membrane transplantation (AMT) was initially considered, it was ultimately deferred due to progressive improvement in visual acuity and ocular surface healing.

Recovery was further complicated by severe oropharyngeal inflammation and painful swallowing, requiring input from speech and language therapy and dietetics to maintain nutrition. Her oral intake and respiratory status gradually improved, and she was discharged on day 21 with tapering ocular therapy and a short course of oral clarithromycin, with follow-up arranged under dermatology and ophthalmology teams.

At the one-month follow-up, the patient demonstrated excellent clinical recovery, with complete resolution of mucocutaneous lesions and improvement in visual acuity (6/9 in the right eye and 6/12 in the left eye), without evidence of conjunctival scarring. Notably, this represents an uncommon favourable outcome, given the severity of ocular involvement in SJS, which is frequently associated with long-term complications. Repeat chest imaging demonstrated the resolution of the previously identified consolidation, and the patient was discharged from both ophthalmology and dermatology follow-up. Blood tests repeated three months after discharge showed complete normalisation of haematological parameters including haemoglobin and neutrophil counts. The transient anaemia during admission was likely multifactorial, reflecting the effects of acute illness, haemodilution from intravenous fluid resuscitation, and repeated phlebotomy, with subsequent recovery on follow-up.

This case demonstrates that even severe levofloxacin-associated SJS with significant ocular involvement can result in favourable clinical outcomes when promptly recognised and managed through coordinated multidisciplinary care.

## Discussion

SJS is a rare, T-cell-mediated hypersensitivity reaction to drugs or their metabolites. Genetic susceptibility, including specific HLA alleles, and immune dysregulation predispose certain individuals; however, only a minority of those exposed to high-risk drugs develop disease [12]. The interaction of a drug-associated antigen or metabolite with major histocompatibility complex (MHC) class I molecules or cellular peptides forms an immunogenic complex [12]. This T-cell-mediated process activates cytotoxic CD8⁺ T lymphocytes, driving keratinocyte apoptosis through several overlapping pathways, including granulysin release (the principal mediator of epidermal necrosis), perforin-granzyme B exocytosis, and Fas-Fas ligand-induced caspase activation [12]. Additional mediators, such as tumour necrosis factor-α and nitric oxide, further amplify keratinocyte death [12]. The combined effect is widespread epidermal necrosis with mucocutaneous involvement, which characterises SJS [12].

In this case, the initial manifestation was swelling of the lips occurring 72 hours after the commencement of levofloxacin, consistent with previously reported cases. Only two published reports have described oral mucosal involvement, including crusting and swelling, developing within 48 hours of levofloxacin administration [8,9]. This case, therefore, underscores the importance of early recognition and management of drug-induced SJS. Although levofloxacin is commonly regarded as a safe alternative in patients with penicillin allergy, it can, in rare instances, precipitate severe cutaneous adverse reactions. Prompt discontinuation of the offending drug remains critical to preventing disease progression.

The patient's SCORTEN score of 3 corresponded to a predicted mortality of approximately 35%, categorising the presentation as high risk [13]. Validated severity assessment tools are essential in SJS to aid prognostication and guide escalation of care [12,13]. The SCORTEN scoring system (the severity-of-illness score for TEN) is a widely used mortality prediction tool in patients with SJS and TEN. It incorporates seven clinical and biochemical parameters: age >40 years, presence of malignancy, heart rate >120 beats per minute, epidermal detachment involving >10% of BSA, serum urea >10 mmol/L, serum glucose >14 mmol/L, and serum bicarbonate <20 mmol/L. Increasing SCORTEN values are associated with a stepwise rise in mortality risk. A score of 0-1 predicts a mortality of approximately 3.2%, 2 12.1%, 3 35.3%, 4 54.3%, and ≥5 approximately 90% [13].

Acute ocular involvement occurs in 50-80% of patients with SJS, and early AMT is widely recommended for moderate to severe disease to prevent long-term sequelae such as symblepharon, scarring, and vision loss [14]. Corneal epithelial defects or pseudomembrane formation, as observed in this case, typically prompt the early consideration of AMT [15]. In contrast, our patient developed bilateral ocular disease, including a 1.7×2 mm corneal epithelial defect in the left eye associated with reduced visual acuity (6/60) and a dendritic ulcer in the right eye, yet achieved complete re-epithelialisation by day 10 and excellent visual recovery at one month (6/9 in the right eye and 6/12 in the left eye). This favourable outcome was achieved without AMT, using intensive topical therapy alone, including preservative-free lubricants, dexamethasone 0.1%, chloramphenicol, and ganciclovir eye drops. No conjunctival scarring or symblepharon developed. Such favourable outcomes in high-risk (SCORTEN 3) SJS cases managed without AMT or systemic immunomodulatory therapy remain uncommon in the literature [15], highlighting the potential for excellent recovery with prompt local treatment and close ophthalmological supervision.

Supportive care remains the cornerstone of SJS management, while evidence for pharmacological therapies remains variable. The benefit of systemic glucocorticoids is inconclusive, and large meta-analyses, including Systemic Immunomodulating Therapies for Stevens-Johnson Syndrome and Toxic Epidermal Necrolysis: A Systematic Review and Meta-Analysis, have not demonstrated a clear survival benefit with intravenous immunoglobulin (IVIG) [16]. Cyclosporine has been associated with improved mortality outcomes in some studies; however, this may partly reflect selection bias, with preferential use in younger patients with fewer comorbidities, and findings should therefore be interpreted with caution [16]. Overall, despite investigation of several immunomodulatory agents, the most reliable predictors of improved outcomes remain early diagnosis, immediate withdrawal of the culprit medication, and high-quality supportive care [16].

Multidisciplinary involvement is essential in the management of SJS. Early dermatology input is crucial for diagnostic confirmation, prognostication using SCORTEN, and guidance on specialist wound care, which is typically conservative and includes sterile water cleansing, emollients, non-adherent dressings, and decompression of tense blisters, with escalation to specialist burn centres in cases of extensive epidermal loss [17]. Ophthalmology involvement is equally vital from the outset, given the high risk of ocular sequelae, with daily review recommended during the acute phase to minimise long-term complications such as symblepharon and blindness. Management is supported with intensive ocular lubrication, topical corticosteroids, and antimicrobial prophylaxis when indicated [15,17]. Speech and language therapy and nutritional support are also important due to frequent oropharyngeal involvement impairing swallowing and oral intake. Depending on disease severity, multidisciplinary care may further involve intensive care, burn services, and dietetics [17].

In addition to the clinical assessment, drug causality was evaluated using the World Health Organization (WHO)-Uppsala Monitoring Centre (UMC) causality assessment system, which classified the association between levofloxacin exposure and the onset of SJS in this case as probable/likely, based on the clear temporal relationship with drug initiation, absence of alternative pharmacological triggers, and clinical improvement following withdrawal of the suspected agent [18].

Collectively, this case underscores the complexity of SJS presentations and the need for tailored, organ-specific management informed by disease severity. Structured multidisciplinary involvement and close clinical surveillance are essential to prevent long-term sequelae associated with severe cutaneous adverse reactions.

## Conclusions

This case highlights that, although fluoroquinolones are an uncommon trigger for SJS, recognition of this potential association is essential to facilitate early diagnosis and timely intervention. Prompt withdrawal of the suspected offending agent and coordinated multidisciplinary supportive care remain central to improving outcomes and can result in favourable clinical recovery, even in high-risk presentations such as those with significant ocular involvement.
